# Functional characterization of acyl-CoA binding protein in *Neospora caninum*

**DOI:** 10.1186/s13071-020-3967-9

**Published:** 2020-02-18

**Authors:** Bingxin Zhou, Yong Fu, Heng Zhang, Xianmei Wang, Gaowei Jin, Jianhai Xu, Qun Liu, Jing Liu

**Affiliations:** 1 National Animal Protozoa Laboratory, College of Veterinary Medicine, China Agricultural University, Beijing, 100193 People’s Republic of China; 2 Key Laboratory of Animal Epidemiology of the Ministry of Agriculture, College of Veterinary Medicine, China Agricultural University, Beijing, 100193 People’s Republic of China

**Keywords:** *Neospora caninum*, Acyl-CoA binding protein, Gene knockout, Fatty acids metabolism

## Abstract

**Background:**

Lipid metabolism is pivotal for the growth of apicomplexan parasites. Lipid synthesis requires bulk carbon skeleton acyl-CoAs, the transport of which depends on the acyl-CoA binding protein (ACBP). In *Neospora caninum*, the causative agent of neosporosis, the FASII pathway is required for growth and pathogenicity. However, little is known about the fatty acid transport mechanism in *N. caninum*.

**Methods:**

We have identified a cytosolic acyl-CoA binding protein, with highly conserved amino acid residues and a typical acyl-CoA binding domain in *N. caninum*. The recombinant NcACBP protein was expressed to verify the binding activities of NcACBP *in vitro*, and the heterologous expression of NcACBP in *Δacbp* yeast *in vivo*. Lipid extraction from *ΔNcACBP* or the wild-type of *N. caninum* was analyzed by GC-MS or TLC. Furthermore, transcriptome analysis was performed to compare the gene expression in different strains.

**Results:**

The NcACBP recombinant protein was able to specifically bind acyl-CoA esters *in vitro*. A yeast complementation assay showed that heterologous expression of NcACBP rescued the phenotypic defects in *Δacbp* yeast, indicating of the binding activity of NcACBP *in vivo*. The disruption of NcACBP did not perturb the parasite’s growth but enhanced its pathogenicity in mice. The lipidomic analysis showed that disruption of NcACBP caused no obvious changes in the overall abundance and turnover of fatty acids while knockout resulted in the accumulation of triacylglycerol. Transcriptional analysis of ACBP-deficient parasites revealed differentially expressed genes involved in a wide range of biological processes such as lipid metabolism, posttranslational modification, and membrane biogenesis.

**Conclusions:**

Our study demonstrated that genetic ablation of NcACBP did not impair the survival and growth phenotype of *N. caninum* but enhanced its pathogenicity in mice. This deletion did not affect the overall fatty acid composition but modified the abundance of TAG. The loss of NcACBP resulted in global changes in the expression of multiple genes. This study provides a foundation for elucidating the molecular mechanism of lipid metabolism in *N. caninum*.

## Background

*Neospora caninum* is an apicomplexan parasite responsible for neosporosis, a disease characterized by abortions and stillbirths (mainly among cattle) and by neuromuscular diseases in dogs [1, 2], that results in substantial economic losses to the beef and dairy industries worldwide [3].

Lipids are crucial to the biology of all cells and organisms, acting not only as primary sources of energy but also as regulators of metabolism and growth, participating in various signaling networks [4]. Lipids are the major structural elements of all biological membranes, serving as signaling molecules within and between cells. As a highly efficient store, the lipids could reduce the energy of all cells and organisms [5, 6]. Lipids can also act as pivotal pathogenetic factors that allow pathogens to escape immune responses, manipulate host processes, and develop disease [7].

Fatty acids provide the acyl skeleton for lipid synthesis. In apicomplexan parasites, the FASII *de novo* synthesis pathway is localized to a special metabolic organelle, the apicoplast, and is responsible for the production of long-chain fatty acids, processed for fatty acids elongation and desaturation in the ER, such as myristic acid and palmitic acid [8, 9]. In addition, parasites can scavenge lipids from the host cells and the surrounding environment [10]. Before entering lipid synthesis pathways, different kinds of acyl-CoA transporters which acts as the major carrier of acyl-CoAs, such as fatty acid binding protein (FABP), sterol carrier protein 2 (SCP2) and acyl-CoA-binding protein (ACBP), would activate and convert fatty acids to fatty acyl-CoA esters *via* a reaction catalyzed by fatty acyl-CoA synthetase and transported to various metabolic locations [11]. ACBP is a protein of approximately 10 kDa that is ubiquitously expressed and highly conserved in tissues with active lipid metabolism such as liver and adipose tissues [12] from humans to protozoans [13] and binds long-chain fatty acid (LCFA) CoA esters (C_14_-C_22_) through its acyl-CoA-binding domains (ACBDs) with high affinity and specificity [11]. Several studies have demonstrated the precise biochemical functions of ACBP, such as binding and transporting acyl-CoAs, maintaining intracellular acyl-CoA pools [14], and participating in membrane biosynthesis [15], fatty acid elongation and sphingolipid synthesis, in eukaryotes [16]. Consistent with its biochemical properties, ACBP deficiency decreases the intracellular LCFA-CoA pool while ACBP overexpression has the opposite effect in yeasts [17] and mice [18]. In addition, it has been shown that ACBP is required for LCFA esterification into triglycerides and phospholipids (PLs) [19, 20] and for oxidation [21]. Furthermore, functional loss of ACBP decreases the very-long-chain fatty acid (VLCFA) content, suggesting that ACBP regulates LCFA elongation and hence VLCFA levels [22].

Functional investigations of ACBPs in apicomplexan parasites have been reported. In *Cryptosporidium parvum*, ACBP is presumed to play roles in lipid metabolism and trafficking in parasitophorous vacuoles (PV) [23]. Recently, our laboratory showed that acyl-CoA binding protein and sterol carrier protein 2 in *Toxoplasma* cooperate in lipid metabolism [24]. As *Neospora* structurally and morphologically resembles *Toxoplasma*, we hypothesized that *Neospora* may depend on the same fatty acid metabolic pathways in which acyl-CoA binding protein determines the metabolic fate of fatty acids. However, little is known about the role of ACBP in *N. caninum.* We identified a candidate gene (NCLIV_066640) encoding a *Neospora* acyl-CoA binding protein *via* bioinformatics analysis. Then, we investigated the expression, localization and activity of this protein *in vitro* and *in vivo*. The combined results of genetic disruption, lipidomic analysis and transcriptome profiling revealed roles of ACBP in lipid metabolism. These data may provide a reference for the further investigation of lipid metabolism in *N. caninum*.

## Methods

### Parasites and cell culture

Human foreskin fibroblasts (HFF, SCSP-106) cells and Vero cells were obtained from the Cell Bank of the Chinese Academy of Sciences (Shanghai, China). The cells were cultured in DMEM (Dulbecco’s modified Eagle’s medium) supplemented with 20% (HFF cells) and 8% (Vero cells) heat-inactivated fetal bovine serum (FBS; Gibco, New York, USA), respectively in a humidified incubator containing 5% CO_2_ at 37 °C. The *N. caninum* wild-type strain (Nc-1) and the constructed NcACBP-deficient strain were cultured as tachyzoites by serial passages in HFF or Vero cells supplemented with 2% FBS, 10 units/ml penicillin and 100 mg/ml streptomycin. The parasites were harvested from freshly lysed Vero cells and washed twice with cold phosphate-buffered saline (PBS), and centrifuged at 1800× *rpm* for 10 min for collection as previously described [25].

### Mice and virulence assay

A virulence assay was performed on six-week-old female BALB/c mice (Peking University Health Science Center, China). The animals were housed under specific-pathogen-free conditions with *ad libitum* access to feed and water. Groups of BALB/c mice (*n* = 5) were infected with 5 × 10^6^ freshly harvested tachyzoites of different strains intraperitoneally.

### Sequence analysis and recombinant protein expression

To obtain detailed information on the acyl-coenzyme A binding protein in *N. caninum*, the *Toxoplasma* genomic resource database (ToxoDB ver.9.0) was used to search for ACBP-related genes. The NcACBP (NCLIV_066640) gene sequence was obtained from the ToxoDB (http://toxodb.org/toxo/) website. Alignment with the corresponding sequence in *Saccharomyces cerevisiae* and in other apicomplexan parasites was performed by Clustal W using DNAMAN (Lynnon Biosoft, San Ramon, USA) and bootstrap consensus trees were then generated. The physical and chemical properties of ACBPs were obtained using the online tool ExPAS-ProtParam (https://web.expasy.org/protparam/); the conserved domains of ACBPs were analyzed by SMART (http://smart.embl-heidelberg.de/) and mapped using IBS 1.0 software.

DNA extraction kit (Aidlab Biotechnologies Co., Ltd., Beijing, China) was used to extract the genomic DNA of the wild-type strain. The open reading frame was amplified by PCR using the primer pairs F1/R1, the 5′UTR, F2/R2, and the 3′UTR, F3/R3. All primers used in this study are listed in Additional file 1: Table S1. Then, we used the pET-28a vector (Novagen, Madison, Germany) to clone the complete coding sequence and transformed into *Escherichia coli* (Transetta, TansGenBiotech Co., Ltd., Beijing, China) for recombinant protein expression. The rNcACBP-His and rNcACBP-GST proteins were purified by affinity chromatography using Ni-IDA agarose in accordance with the manufacturer’s standard protocol. Purified recombinant proteins were assessed by SDS-PAGE analysis. The gel was consisted of running gel (12%, w/v, acrylamide) and stacking gel. The electrophoresis buffer was 25 mM Tris, 192 mM glycine, 0.1% SDS, pH 8.3. After electrophoresis, the gels were stained using Coomassie brilliant blue (Thermo Fisher Scientific Inc., Waltham, MA, USA) for protein detection. They were treated in protein destaining solution (10% acetic acid, 30% methyl alcohol, 60% distilled water, v:v:v) overnight. The relative molecular mass of each of the recognized bands was determined by comparison with standard markers.

### Quantitative real-time PCR (qRT-PCR)

Total RNA was extracted from 1 × 10^8^ tachyzoites of the wild-type strain and *ΔNcACBP* strain with TRIzol reagent and converted to cDNA using an EasyScript First-Strand cDNA Synthesis SuperMix kit (TransGen, Beijing, China) in accordance with the manufacturer’s instructions. The NcActin (NcLiv_061190) gene was selected as the endogenous reference gene to normalize the *P*-value in each sample [26]. As per the manufacturer’s instructions, RT-PCR was performed in triplicate with three independent samples for each experimental group in an ABI Prism 7500 System (Applied Biosystems Inc., Foster, USA) with SYBR Green II (Takara Biotechnology, Dalian, Co., Ltd, China). The RT-PCR conditions were as follows: 94 °C for 5 s, followed by 40 cycles at 94 °C for 5 s and 60 °C for 30 s. The relative expression levels of genes were calculated from the quantification cycle (Cq) value and standardized by the 2^−ΔΔCq^ method [27]. All primers used in this study are listed in Additional file 1: Table S1.

### Enzyme kinetics and substrate preference assays

The *in vitro* binding kinetics and substrate preference of the rNcACBP-GST protein were determined by an NBD-C16:0-CoA-based assay as described previously [23, 28]. The reaction components consisted of 0.25 μM fluorescently labeled NBD-C16:0-CoA substrate, 1 μM rNcACBP-GST or GST protein (as the negative control group), and PBS in a final volume of 100 μl. The enzyme kinetic assays were performed using 1 μM rNcACBP-GST protein and NBD-C16:0-CoA (0–0.3 μM) and PBS in a final volume of 100 μl. The reactions were performed in a 96-well plate and incubated for 5 min at room temperature to ensure maximum binding before proceeding with fluorescence measurements. The fluorescence intensity of the conjugates was measured at 460 nm and 538 nm by a SpectraMax M5.

### Heterologous complementation of the yeast ACBP gene by the NcACBP gene

To determine whether the NcACBP gene could complement the yeast ACBP gene, the NcACBP gene was inserted into the yeast expression vector p405ADH1 under the control of the ADH1 promoter and CYC1 terminator. The yeast wild-type and ACBP null mutant (*ΔScACBP*) strains (Dharmacon Inc., Lafayette, CO, USA) were obtained and grown on YPD (1% yeast extract, 2% peptone, 2% dextrose) medium. 2% agar was added to obtain a solid medium if necessary. To express *N. caninum* proteins in a yeast strain defective in ScACBP formation, the putative NcACBP sequence was released from the p405ADH vector by restriction digestion with *BamH*I and *Xho*I, purified and ligated into the p405ADH-NcACBP plasmid digested with *Sac*I. The linearized p405ADH-NcACBP plasmid was transformed into the *ΔScACBP* strain using a commercial kit (FunGenome Company, Beijing, China) according to the manufacturer’s instructions. Candidate transformants were picked and streaked on plates lacking leucine (SCGal-Glu-Leu) to select against the presence of the p405ADH-NcACBP plasmid. Analysis of the yeast vacuolar structure was performed using the fluorescent vital dye FM4-64 [29, 30], and visualized by a Leica confocal microscope system (Leica TCS SP52, Wetzlar, Germany).

### Immunofluorescence assay

The subcellular localization of NcACBP and apicoplast were detected by IFA. Tachyzoites that freshly released or infected HFF cells were fixed with 4% paraformaldehyde for 30 min, as previously described [26]. Samples were blocked with 3% BSA-PBS after permeabilized with 0.1% Triton X-100 and incubated with primary antibodies for 1 h. Rabbit anti-NcSRS2 (1:500), mouse anti-HA (1:500), mouse anti-NcENR (1:500) were used as primary antibodies in this study. Then, FITC-conjugated goat-anti mouse IgG (Sigma-Aldrich, Louis, MO, USA) and Cy3-conjugated goat-anti-rabbit IgG (Sigma-Adrich) were used as secondary antibodies at 1:1000 dilution for labeling. The nuclear was stained with Hoechst (1:100) (Sigma-Aldrich), and the lipid bodies were stained with Nile red (1:50). Mouse anti-HA monoclonal antibody was purchased from Sigma-Aldrich. Mouse anti-NcENR and rabbit anti-NcSRS2 were all polyclonal antibodies stored in our laboratory.

### Construction of the NcACBP knockout *N. caninum* strain

We used the homologous recombination strategy and a CRISPR/Cas9 plasmid to construct the NcACBP deletion strain. The pTCR-CD plasmid contains the chloramphenicol resistance gene (CmR), red fluorescence protein gene (RFP), bacterial cytosine deaminase gene (CD), and ampicillin resistance gene (Amp), and was modified as previously described [31]. The expression of the CmR-RFP fusion gene and CD gene were under the control of the NcTublin promoter. The NcACBP 5′ and 3′ untranslated region (UTR) fragments were amplified and inserted into the pTCR-CD plasmid to delete the NcACBP gene. To produce the NcACBP gene deletion plasmid, correctly sequenced plasmids were double digested with *Hind*III and *Xho*I for the 5′ UTR and with *Xma*I and *Spe*I (NEB, Ipswich, USA) for the 3′ UTR, named pTCR-NcACBP-CD KO. The linearized pTCR-NcACBP-CD KO plasmid, digested with *Not*I (NEB), was purified using ethanol precipitation and then resuspended by cytomix [32]. The single guide RNA (5′-GCT TAC AAA AGG CTC ATT CA-3′) was designed using the E-CRISPR website to recruit Cas9 and cut the NcACBP gene. In addition, 19-bp sequences, up- and downstream in the CRISPR/Cas9 plasmid were selected as overlapping regions. Correctly sequenced CRISPR/Cas9 plasmids were double digested with *Kpn*I and *Avr*II to produce the NcACBP gene deletion plasmid, named CRISPR/Cas9-NcACBP. All constructs were verified by sequencing (Beijing Ruibiotech Co. Ltd, Beijing, China). Fifty μg of the above plasmids were electroporated into wild-type tachyzoites (1 × 10^7^). The transgenic parasites were grown under chloramphenicol (20 mM) and 5-fluorocytosine (40 mM) selection pressure and then screened to confirm the purity of the selected strains until cultured consecutively to the 10th generation.

### Plaque assay

The size and number of plaques represent parasites’ successive rounds of lytic cycles, including invasion, replication, and egress, and thus can be used to evaluate the exhaustive fitness of tachyzoites. HFF cells were seeded in 6-well plates previously, then infected with 800 parasites per well and incubated for 7 days in a 37 °C incubator with 5% CO_2_. Subsequently, cells were stained with 2% crystal violet for 15 min after fixed in PBS containing 4% paraformaldehyde for 30 min. The stained wells were washed with deionized water, airdried and visualized by microscopy (Olympus Co., Tokyo, Japan) using image acquisition software. Plaque area was measured using photoshop by statistical pixels [31].

### Proliferation assay

HFF cells were prepared into 24-well plates with coverslips before infected with 800 tachyzoites per well and cultured at 37 °C in DMEM with 20% FBS for 30 min, then washed with PBS for three times. Then cells were washed with PBS and fixed with 4% paraformaldehyde after adhered overnight in a 37 °C incubator with 5% CO_2_, followed by IFA assay to counted the number of parasites per vacuole with a fluorescence microscope.

### GC-MS analysis

Intracellular tachyzoites (1 × 10^8^) were harvested and total lipids were extracted in chloroform/methanol (1:2, v/v) for 30 min at 60 °C, as previously described [33, 34]. Then, polar and nonpolar metabolites were separated by phase partitioning. The organic phase was dried under N_2_ gas and dissolved in chloroform/methanol (2:1, v/v) for lipid analysis. Then, the lipids were mixed with 1 nM lauric acid (C12:0) as the internal standard and derivatized using MethPrep II (Alltech, Chicago, Illinois, USA). The resulting fatty acid methyl esters were analyzed using GC-MS [33, 35]. The comparison of retention times and mass spectra from GC-MS could identify all the fatty acid methyl esters profile with authentic chemical standards. The data were analyzed with the Agilent workstation software MSD Chemstation D.01.02.16 and compared with the spectral library. The peak areas of fatty acids from *N. caninum* were analyzed and standardized with respect to the internal standard. Finally, the peak area ratio of total fatty acids from different strains was calculated.

### Stable isotope metabolic labeling of *N. caninum* fatty acids

Stable isotope metabolic labeling experiments followed by lipid extraction and GC-MS analysis were performed as previously described [33–35]. Infected HFF cells were cultured in medium in which the unlabeled glucose was replaced with 8 mM U-13C-glucose (Cambridge Isotope Laboratories, Andover, MA, USA) for polar metabolites 24 h before the tachyzoites egress. All lipids were analyzed by GC-MS after derivatization using MethPrep II (Alltech). The shift in the mass spectrum of each fatty acid was analyzed to assess the incorporation of ^13^C into fatty acids.

### Thin layer chromatography

We adopted the Folch method to extract the total lipids as previously described [36]. In short, fatty acids were released as their methyl esters after treated with chloroform/methanol (2:1 v/v) and dried under N_2_. Each sample was suspended in 50 μl of chloroform, and 10 μl was loaded onto TLC plates (Whatman, Maidstone, Kent, UK). The samples were delivered by small drops, and the esterification mix was loaded as the standard marker to quantify total palmitate. On TLC Silica Gel 60 plates (Merck, Gibbsboro, NJ, USA), total lipids were separated by a solvent for neutral lipids (hexane: diethyl ether: acetic acid (90:10:1, v/v/v)) and run with lipid standards for the separation of total phospholipids from mono-, di-, and triacylglycerols (TAG). Canon digital scanner (model F917500; Tokyo, Japan) was used to image the plates, and the intensities of TAG bands were measured by densitometry.

### Lipid body staining

We used fluorescence microscopy to detected the lipid bodies in *N. caninum*, intravacuolar parasites were fixed in 4% paraformaldehyde and following the IFA protocol. Lipid bodies were stained with Nile red [37], a fluorescent dye that preferentially binds neutral lipids such as triglycerides to assess the effect of NcACBP on lipid accumulation. Infected cells and extracellular tachyzoites were fixed with 4% paraformaldehyde, and permeabilize with 0.1% Triton X-100 for 15 min, then incubated with the Nile red in the dark at RT for 20 min. Cells were washed in PBS and imaged using a Leica confocal microscope system (TCS SP52; Leica, Wetzlar, Germany).

### RNA-seq analysis

Transcriptome sequencing was performed according to the manufacturer’s recommendations. Briefly, a total amount of 3 μg RNA per sample was used as input material for the RNA sample preparation and subjected to poly-T oligo-attached magnetic bead enrichment. Sequencing libraries were generated using the NEBNext® Ultra^TM^ Directional RNA Library Prep Kit (Illumina, NEB, Ipswich, USA) following the manufacturer’s instructions and index codes were added to attribute sequences to each sample. Library quality was assessed on the Agilent Bioanalyzer 2100 system. The clustering of the index-coded samples was performed on a cBot Cluster Generation System using TruSeq PE Cluster Kit v3-cBot-HS (Illumina), according to the manufacturer’s recommendations. After cluster generation, the library preparations were sequenced on an Illumina Hiseq platform and paired-end reads were generated. All treatments and subsequent analyses were performed on individual transcripts.

### Differential expression analysis

Differential expression analysis was performed using the DESeq R package (1.18.0). DESeq provides statistical routines for determining differential expression in digital gene expression data using a model based on the negative binomial distribution. Raw counts were normalized using the Benjamini and Hochberg’s approach for controlling the false discovery rate (FDR) [38]. A corrected *P*-value of 0.005 and log_2_(Fold change) of 1.2 were set as the threshold for significantly differential expression [39, 40].

### GO and KEGG enrichment analysis of differentially expressed genes

Gene Ontology (GO) enrichment analysis of differentially expressed genes was implemented by the GOseq package in R software [41], in which gene length bias was corrected. GO terms with a corrected *P*-value less than 0.05 were considered significantly functional enrichment in differential expressed genes.

KEGG is a database resource for understanding high-level functions and utilities of the biological system, such as the cell, the organism and the ecosystem, from molecular-level information, especially large-scale molecular datasets generated by genome sequencing and other high-throughput experimental technologies (http://www.genome.jp/kegg/) [42]. We used KOBAS software to test the statistical enrichment of differential expression genes in KEGG pathways.

### Statistical analysis

Graphs and statistical analyses were made using GraphPad Prism (GraphPad, San Diego, CA, USA). All data were analyzed using Student’s t-test and univariate analysis of survival using Log-rank (Mantel-Cox) test. *P*-values are represented in the figures as follows: *P < 0.05; **P < 0.01; ***P < 0.001; ns, not significant.

## Results

### NcACBP is a conserved protein in apicomplexan parasites

**Fig. 1:**
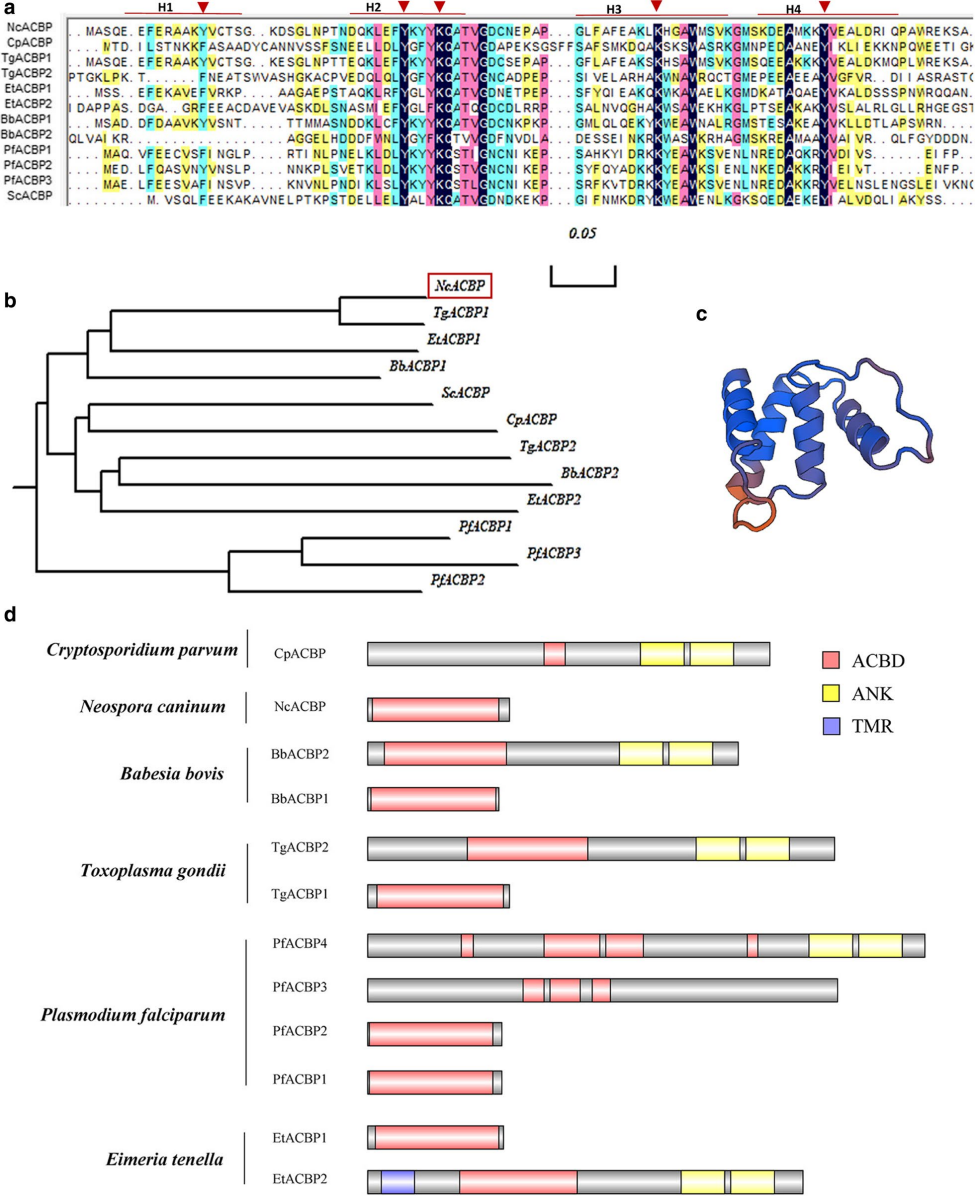
Sequence analysis and expression of NcACBP. **a** Sequence alignment of the ACBPs from *N. caninum*, *S. cerevisiae* and other apicomplexan species. The colored background, black, pink, blue and yellow indicates 100%, 75%, 50% and 30% conserved residues, respectively. The red inverted triangles indicate the potential binding sites for acyl-CoA esters, and H1-H4 indicates the positions of four putative alpha-helices. **b** Phylogenetic analysis of ACBP gene family evolution in apicomplexan classes and *S. cerevisiae*. **c** Schematic domain structures of NcACBP. **d** Schematic domain structures of the apicomplexan ACBPs. *Abbreviations*: ACBD, acyl-CoA-binding domain; ANK, ankyrin repeats; TMR, transmembrane region. *Note*: GenBank accession numbers and/or loci: NcACBP (CBZ56239.1); CpACBP (ABD65295.1); TgACBP1 (EPR63321.1); TgACBP2 (EPR61883.1); PfACBP1 (CDO67240.1); PfACBP2 (XP_001347301.1); PfACBP3 (XP_001347300.2); PfACBP4 (CZT98852.1); BbACBP1 (EDO05649.1); BbACBP2 (BAN65861.1); EtACBP1 (XP_013234417.1); EtACBP2 (XP_013229650.1); ScACBP (AAA34384.1)

Only one protein containing the acyl-coenzyme A binding domain was found on chromosome XII in *N. caninum*, also named the diazepam binding inhibitor based on the annotations. Multiple alignments performed by Clustal W showed there were many highly conserved amino acid residues between *S. cerevisiae* and other apicomplexan species which were reported to play important roles in binding acyl-CoA ligand [11]. The conserved Lys residues in H2 and H3 α-helices clusters and Tyr residue in H2 are presumed to interact with 3′-phosphate group of the CoA part in the acyl-CoA ligand, while the conserved Tyr in H4 may contribute to the stacking of its own aromatic ring and the adenine ring of the CoA part of ligand. The triangles indicate the potential binding sites for acyl-CoA esters, and H1-H4 indicates the positions of four putative alpha-helices (Fig. 1a). TgACBP1 shares the highest amino acid sequence identity (85.26%) with NcACBP by phylogenetic analysis (Fig. 1b). NcACBP consists of 95 amino acids and its predicted molecular weight is ~ 10.7 kDa. The 3D structure of NcACBP showed four α-helix bundles that constitute the binding pocket for acyl-CoA esters (Fig. 1c). In the large molecular weight ACBPs (> 12 kDa), the conserved acyl-CoA-binding domain (ACBD) lies at the N-terminus while other structural domains, such as the ankyrin repeats (ANK) domain, reside at the C-terminus, and some of these proteins contain a transmembrane region (TMR) at the N-terminus (i.e. EtACBP2). In contrast, the small molecular weight ACBPs (10-kDa) contain only one conserved acyl-CoA-binding domain (Fig. 1d). These data indicate that NcACBP is a conserved protein in apicomplexan parasites.

### Identification and cellular localization of NcACBP

**Fig. 2:**
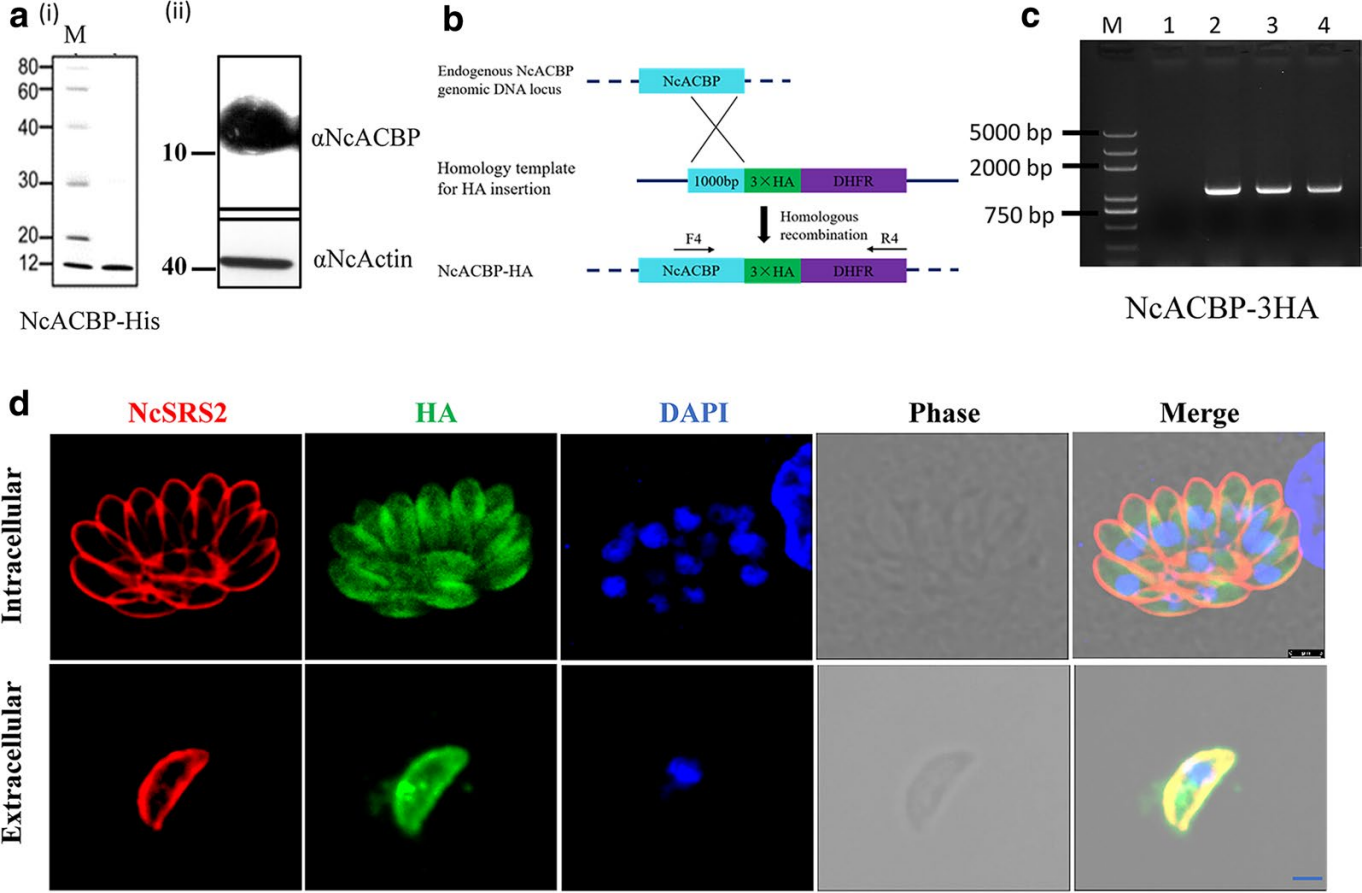
Identification and cellular localization of NcACBP. **a** Expression of NcACBP in *E. coli* (**i**) and in *N. caninum* (**ii**). **b** The schematic design of NcACBP endogenously tagged with HA at its C-terminal. The HA tag of NcACBP is fused using single homologous recombination strategy. **c** Identification of NcACBP-3HA. Lane 1: parental strain Nc-1 as the template; Lanes 2, 3, 4: three clones of NcACBP-HA as the template, respectively. **d** Location of the NcACBP. NcACBP, stained with mouse anti-HA antibody, was distributed in the cytoplasm of intracellular and extracellular parasites. NcSRS2 was used as a marker to indicate the outlines of parasites, and nuclear DNA was stained with Hoechst (blue). *Scale-bars*: 1 μm

To assess the expression and localization of NcACBP, recombinant proteins fused with a GST tag and/or histidine tag were expressed in *E. coli* successfully. The recombinant protein rNcACBP-His was identified by SDS-PAGE (Fig. 2a-i). The native NcACBP expression in *N. caninum* was identified by western blot. The expected bands were elicited by anti-NcACBP polyclonal antibody, recognized an ~ 11 kDa protein in the lysate of tachyzoites and NcActin served as the loading control (Fig. 2a-ii). To localize the NcACBP, the C-terminal of NcACBP was fused with a triple hemagglutinin (3× HA) epitope tag by single homologous recombination (Fig. 2b). The IFA showed that NcACBP was distributed in the cytosol of intracellular and extracellular parasites (Fig. 2d), suggesting that NcACBP is a cytoplasmic protein in *N. caninum*.

### Determination of NcACBP binding activity *in vitro* and *in vivo*

**Fig. 3:**
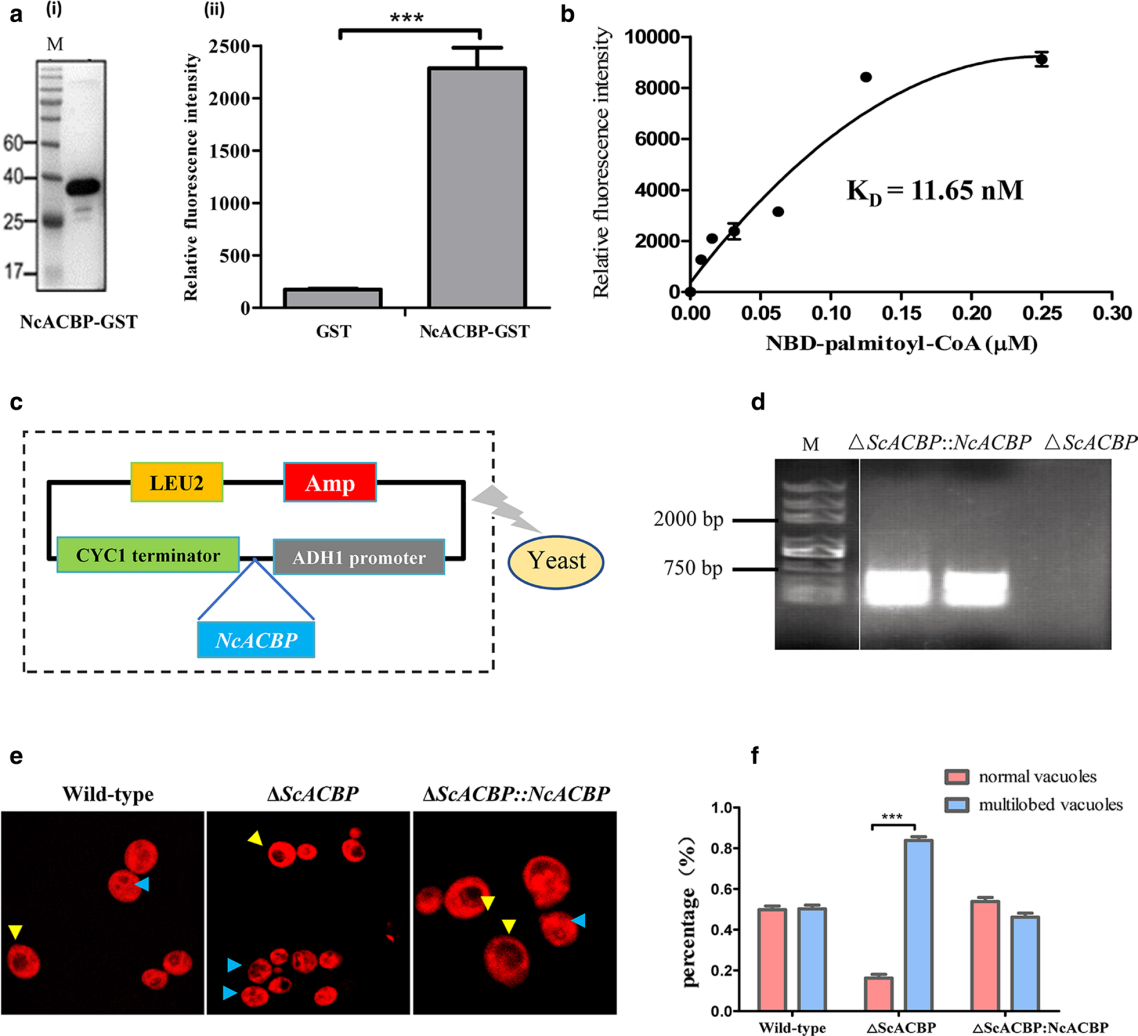
NcACBP functions as an active acyl-CoA-binding protein *in vitro* and *in vivo*. **a** Acyl-CoA binding activity of NcACBP. The SDS-PAGE analysis of purified recombinant protein NcACBP-GST from *E. coli* (**a**-**i**). Acyl-CoA binding activity of NcACBP was confirmed by incubating of the fluorescent substrate NBD-palmitoyl-CoA (0.25 μM) with GST-NcACBP (**a**-**ii**). **b** The binding kinetics of GST-NcACBP with NBD-palmitoyl-CoA were determined *via* a fluorescence assay. **c** Schematic illustrating the genetic complementation of *ΔScACBP* mutant yeast. **d** NcACBP gene (288 bp) can be detected in *ΔScACBP:NcACBP* clones. Lane 1 and lane 2 were different clones of *ΔScACBP:NcACBP*; lane 3 was *ΔScACBP* mutant yeast. **e** Phenotype rescue in yeast. Representative micrographs of wild-type *S. cerevisiae* yeast, *ΔScACBP* mutant yeast and *ΔScACBP:NcACBP* yeast were shown. Yeasts with single- and multilobed vacuoles were shown by yellow and blue arrowheads, respectively. **f** Yeast cells were categorized as normal or multilobed based on the phenotype. The bars indicate the means ± SD

To verify the binding activities of NcACBP *in vitro*, we expressed recombinant NcACBP-GST in *E. coli* and performed fluorescent substrate binding assay using NBD-C16:0-CoA. We observed an increased fluorescence upon the binding of NBD-C16:0-CoA to rNcACBP, while there is no signal in the GST control group (Fig. 3a). Through this fluorometric assay, we determined the dissociation constant (K_D_) of NcACBP 11.65 nM for NBD-C16:0-CoA (Fig. 3b). These results indicate that rNcACBP has an acyl-CoA binding activity *in vitro*. Additionally, the function of NcACBP *in vivo* was analyzed. The NcACBP gene was inserted into the yeast expression vector p405ADH1 under the control of the ADH1 promoter and CYC1 terminator and transforming into *ΔScACBP* mutant yeast (Fig. 3c–d). Transformed yeast cells were stained with the vacuolar marker FM4-64 and observed with a fluorescence microscope. Lack of ScACBP caused disintegration of yeast vacuoles, leading to an increased number of cells with the multilobed vacuole phenotype. Cells transformed with an empty vector did not alter the vacuolar disintegration phenotype; however, yeast cells expressed NcACBP protein complemented the phenotype (Fig. 3e–f). The significance was determined by Student’s t-test: *t*_(5)_ = 7.481, *P*  < 0.001. These results demonstrated that NcACBP can bind acyl-CoA *in vitro* and complement the function of yeast ACBP *in vivo*.

### NcACBP is not essential during the tachyzoite stage

**Fig. 4:**
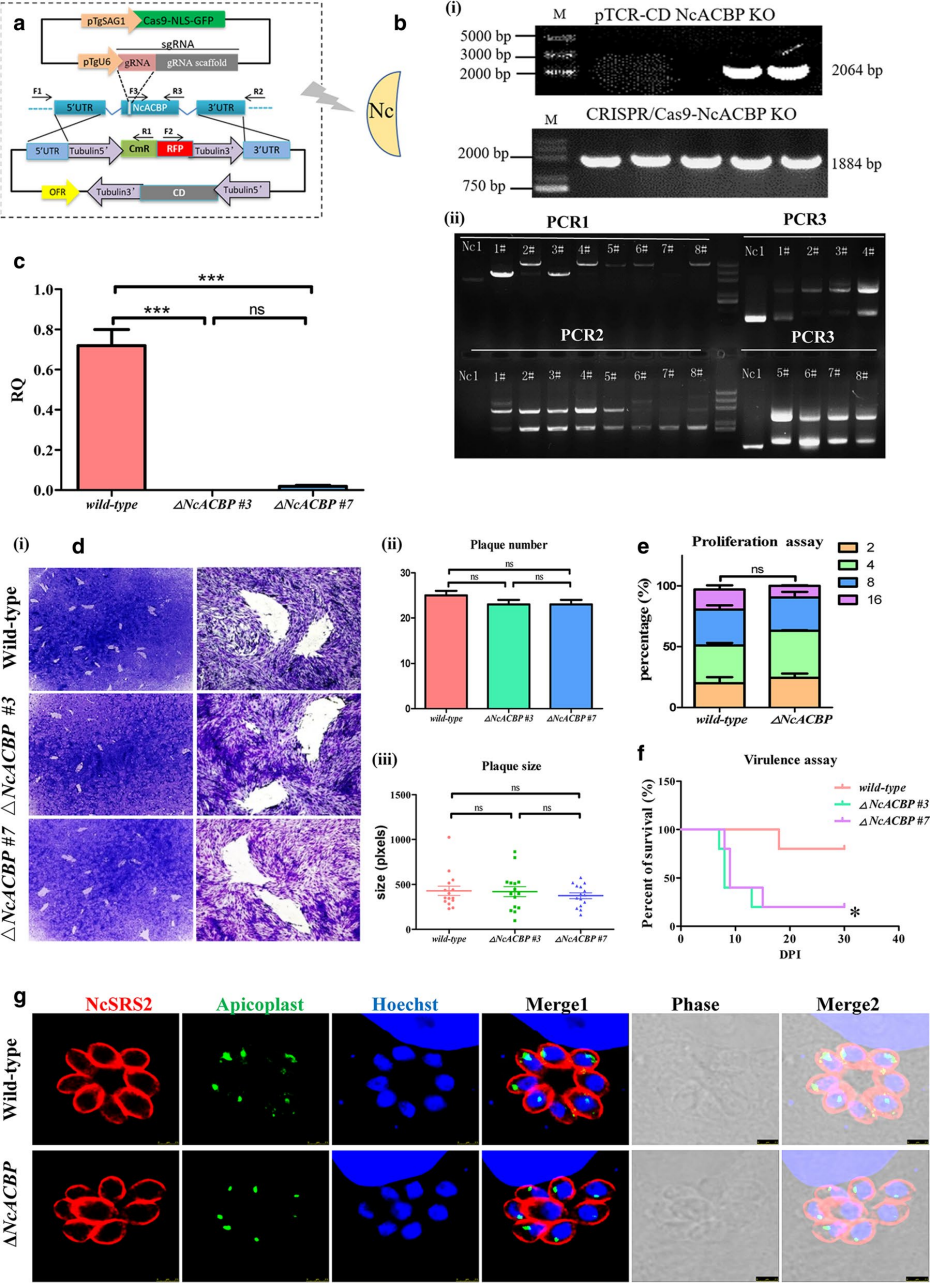
Deletion of the NcACBP gene did not affect parasite morphology, virulence or apicoplast biogenesis. **a** Schematic illustration of the NcACBP knockout. **b**-**i** Identified the knockout plasmids by PCR; each lane represents a different clone. **b**-**ii** The genomic PCR identification of the *ΔNcACBP* strain. The position of the primers was shown in the pattern diagram. The numbers #1-#8 represent different clones. **c** Quantitative RT-PCR was used to analyze the transcription levels of the NcACBP gene in the *ΔNcACBP* clones and in the wild-type. **d** Plaque assay comparing the growth of *ΔNcACBP* clones and wild-type parasites. The growth ability of parasites was evaluated by the number of plaques (**d**-**ii**) and the plaque sizes (**d**-**iii**). **e** Intracellular parasite replication of *ΔNcACBP* was compared with wild-type. Data were compiled from three independent assays, and 100 total PVs of each strain were counted in each assay. **f** Mouse survival after infection with *ΔNcACBP* or Nc-1. BALB/c mouse (*n* = 5) were injected i.p. with 5 × 10^6^ parasites. The data were representative of three experiments with similar outcomes. **g** Detection of apicoplasts in the Nc-1 and *ΔNcACBP* strains. Apicoplasts were stained with mouse anti-NcENR antibodies. *Scale-bars*: 2.5 μm

To characterize the role of NcACBP in *N. caninum*, we generated a complete knockout mutant of NcACBP (*ΔNcACBP*) *via* the CRISPR/Cas9 system. By targeting the native NcACBP locus in the wild-type strain, the gene was replaced by the CAT-RFP cassette through homologous recombination (Fig. 4a). The deletion of ACBP gene was confirmed by PCR (Fig. 4b). The RT-PCR results also showed that the transcriptional level of NcACBP was significantly reduced after deletion (Fig. 4c). The phenotype assays showed that knockout of NcACBP did not affect the plaque number and size (Fig. 4d) and did not affect the replication of parasites either (Fig. 4e). These data showed that NcACBP is not an essential gene in *N. caninum* during the tachyzoite stage.

### Knockout of NcACBP enhanced the pathogenicity of *N. caninum* in mice

To evaluate the effects of NcACBP on *N. caninum* pathogenicity, BALB/c mice were intraperitoneally infected with 5 × 10^6^ freshly released tachyzoites of the wild-type or *ΔNcACBP*. Signs of illness, such as coat ruffling, inactivity, and mental depression, were observed after 4 days post-infection. The mice infected with the *ΔNcACBP* strain began to die at 8 days post-infection, while mice infected with the wild-type strain showed a significant delay in the time of death (dpi = 18). The survival rate of *ΔNcACBP* infected mice was lower than the wild-type infected mice significantly. Significance was determined by Log-rank (Mantel-Cox) test: *χ*^2^ = 9.67, *df* = 15, *P* = 0.0146 (Fig. 4f). These results demonstrate that the pathogenicity of *N. caninum* in mice was enhanced after the deletion of NcACBP.

### NcACBP disruption does not impair the biogenesis of the apicoplast

Since ACBPs were indicated to participate in acyl-CoA binding and transport, the maintenance of intracellular acyl-CoA pools and membrane biosynthesis in eukaryotes, it is possible that loss of NcACBP may affect the synthesis of fatty acids. To determine the potential role of NcACBP in fatty acid synthesis, especially *de novo* fatty acid synthesis in the apicoplast, the apicoplast was stained with mouse anti-NcENR antibodies, which showed that disruption of NcACBP did not affect the morphology of the apicoplast (Fig. 4g), demonstrating that loss of NcACBP does not impair the biogenesis of the apicoplast during the tachyzoite stage.

### NcACBP disruption does not affect the total fatty acid composition

**Fig. 5:**
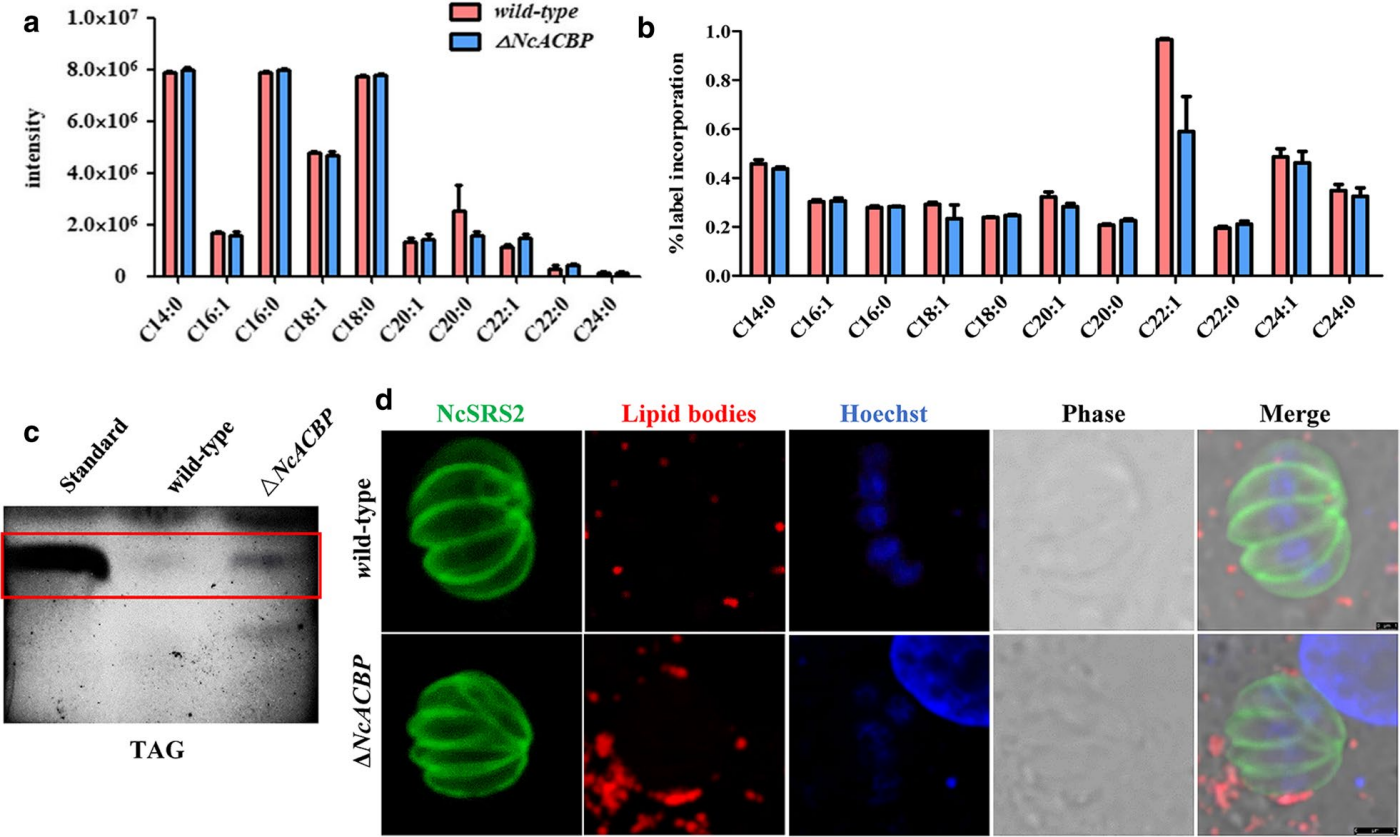
Roles of NcACBP in parasite lipid metabolism. **a** The abundance of fatty acids in *ΔNcACBP* and wild-type parasites was determined by GC-MS. Total lipids were extracted from each strain, followed by derivatization with MethPrep II to yield fatty acid methyl esters, and the lipid amounts were quantified by GC-MS following normalization according to an internal standard (C14:0) and cell numbers. **b** The incorporation of ^13^C into each fatty acid species is shown. The bars show the means of three technical replicates, and the error bars indicate the standard deviations of these measurements. **c** Total lipids were extracted from tachyzoites and separated by TLC. Neutral lipids were separated on silica gel plates. Triacylglycerol is indicated by the red rectangle compared with the TAG standard. **d** Host lipid bodies were observed using Nile red staining. NcSRS2 was used to indicate the outlines of parasites, and the nuclear DNA was stained with Hoechst. *Scale-bars*: 1 μm

To validate our hypothesis that loss of the NcACBP may alter the lipid profile in parasites, the fatty acids were extracted from both the wild-type and *ΔNcACBP* strains and its species and abundance were analyzed by GC-MS. The results showed that there was no obvious difference in the total fatty acid content between the parental and *ΔNcACBP* strains (Fig. 5a), indicating that disruption of NcACBP did not significantly affect the abundance of fatty acids. To investigate the effect of disruption of NcACBP on the fatty acid synthesis, we labeled parasites with U-^13^C-glucose as previously reported [11]. The GC-MS results showed that this disruption did not cause any changes in the incorporation of ^13^C into fatty acids (Fig. 5b). These data demonstrate that NcACBP is dispensable for fatty acid production.

### NcACBP disruption leads to the accumulation of neutral lipids in parasites

To verify whether disruption of NcACBP affects lipid accumulation, we extracted lipids from wild-type and *ΔNcACBP* parasites and performed TLC analysis, which showed an increased abundance of TAG in *ΔNcACBP* parasites compared to that of parental parasites (Fig. 5c). Then, we focused on the delivery of neutral lipids stored in host lipid bodies to the PV and the parasite, as several pathogens are able to recruit lipid bodies around their vacuoles [43, 44]. The infected cells were incubated with Nile red, a fluorescent dye that preferentially binds neutral lipids such as triglycerides. Compared with cells infected with the wild-type strain, numbers and size of the lipid bodies were increased in HFF cells infected with *ΔNcACBP* strains; however, there was no statistically significant difference (Fig. 5d).

### Loss of NcACBP results in global changes in the expression of multiple genes

**Fig. 6:**
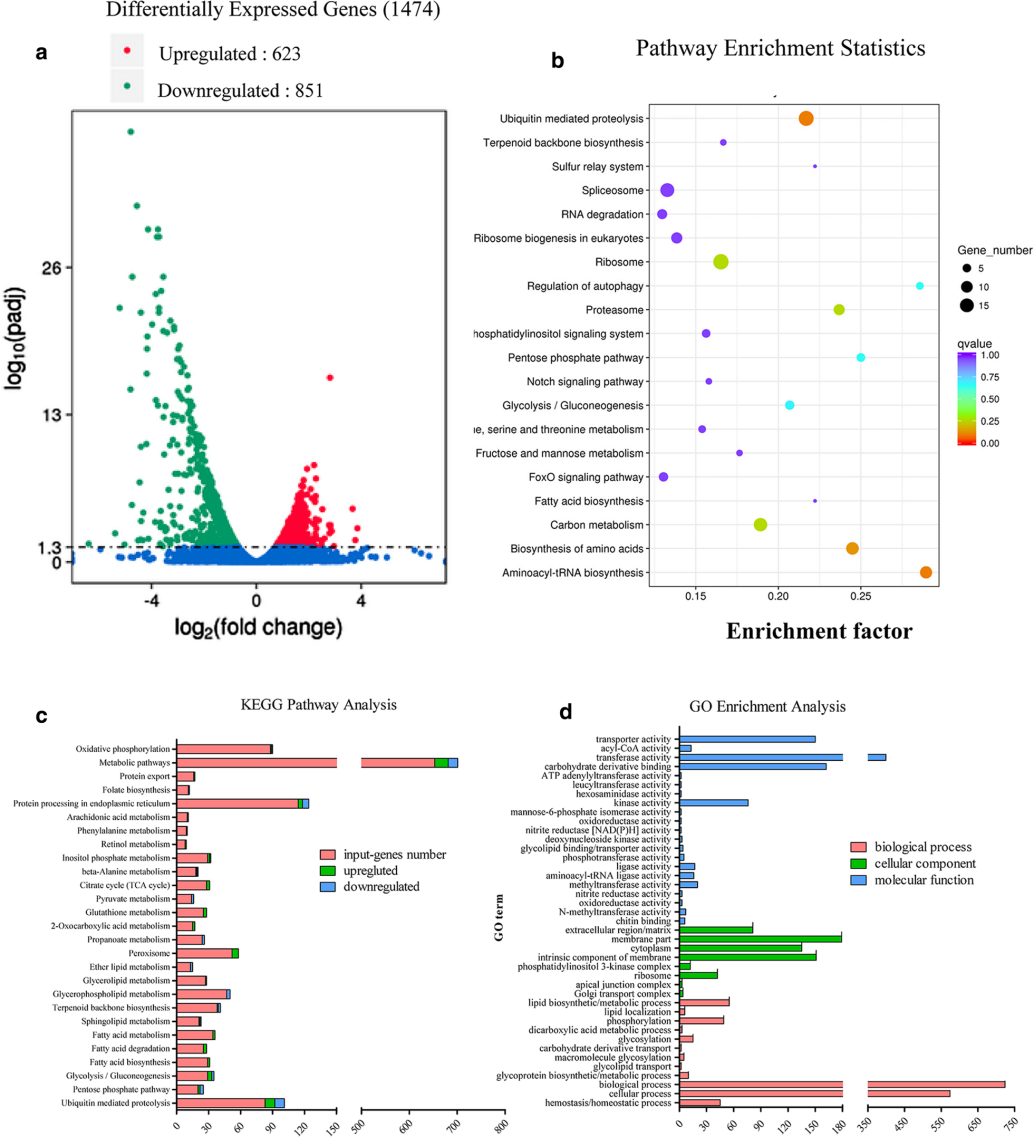
Loss of NcACBP globally alters the expression of multiple genes. **a** Differentially expressed (log_2_ fold change ≥ 1.2) genes analysis of a total of 1474 genes compared *ΔNcACBP* strain to the wild-type. Downregulated genes (*n* *=* 851) are highlighted in green, and upregulated genes (*n* *=* 623) are highlighted in red. **b** Scatter plot of pathway enrichment analysis of differentially expressed genes between *ΔNcACBP* and wild-type parasites. **c** KEGG pathway analysis of 1474 differentially expressed genes in *ΔNcACBP*. Classifications were manually assigned according to known or putative functions. **d** GO enrichment analysis of differentially expressed genes between *ΔNcACBP* and wild-type parasites. Differentially expressed genes were divided into three groups: biological process, cellular component and molecular function

Transcriptome analysis was performed to compare the gene expression levels in wild-type and *ΔNcACBP*, so as to investigate the mechanism of the phenotypic changes in *ΔNcACBP*. Statistical analysis showed that 1474 genes were expressed differentially, including 623 upregulated genes and 851 downregulated genes with a log_2_ fold change of ≥ 1.2 (Fig. 6a) [45]. The regulated genes with a log2 fold change of ≥ 4 between Nc-1 and *ΔNcACBP* strains are provided in Additional file 1: Table S2. To characterize 1474 differentially expressed genes, Kyoto Encyclopedia of Genes and Genomes (KEGG) pathway analysis was executed. The most highly enriched canonical pathways were the ubiquitin-mediated proteolysis, aminoacyl-tRNA biosynthesis, glycolysis and gluconeogenesis, proteasome, ribosome and regulation of autophagy pathways (Fig. 6b). We also found several differentially expressed genes involved in multiple metabolic pathways, including oxidative phosphorylation, peroxisome, protein processing in the endoplasmic reticulum and glycerophospholipid metabolism pathways. Several genes involved in fatty acid biosynthesis and metabolism were upregulated, while genes involved in ER protein processing and glycerophospholipid metabolism were downregulated (Fig. 6c). These results suggest that loss of NcACBP affects the metabolism of fatty acids and glycerol phospholipids in *N. caninum* and results in global changes in the expression of multiple genes involved in various biological functions and cell components. Gene Ontology enrichment analysis was executed to identify the functions of the differentially expressed genes. These genes were classified into three categories: biological process, cellular component and molecular function. We observed that 1344 genes were involved in biological processes, such as glycoprotein biosynthetic and metabolic processes, cellular lipid biosynthesis and metabolic processes, phosphorylation and glycosylation; 621 genes participated in molecular function (i.e. transferase activity, transfer pentosyl groups, kinase activity and transporter activity); and 331 genes took part in cellular components, particularly in the components of extracellular region, membrane region and cytoplasm (Fig. 6d). These data demonstrate that the deletion of NcACBP damages the profile of the cell membrane, the metabolism of glycoproteins and lipids, phosphorylation and glycosylation.

## Discussion

ACBP, originally identified as a mammalian diazepam-binding inhibitor [46], mainly functions as an intracellular acyl-CoA transporter and pool former [11] and is critical to lipid metabolism in cells [47]. Among apicomplexans, NcACBP functions as a short protein with a unique acyl-CoA binding domain and is dispersed in the cytosol of either intracellular or extracellular parasites. However, other apicomplexans may have multiple ACBP proteins of various types (i.e. short proteins or long proteins fused with ankyrin repeats or with containing transmembrane region domains), revealing that ACBP-mediated metabolic pathways may be highly divergent in the phylum Apicomplexa. Our biochemical data indicate that rNcACBP is capable of binding to palmitoyl-CoA (25 μM) with the highest binding affinity (K_D_ = 11.65 nM) *in vitro*. In addition, the abnormal phenotype of slow growth and an increased number of multilobed vacuoles in *ΔScACBP* mutant yeast [15] can be rescued by NcACBP, similar to the effect of RpACBP-1 [48], demonstrating that NcACBP possibly plays a similar role to that of ScACBP as a homologous protein.

Although the deletion of NcACBP by CRISPR/Cas9 using homologous recombination did not perturb the survival and morphology of the parasites *in vitro*, its pathogenicity in mice was enhanced. We have compared the transcriptomes between *ΔNcACBP* and wild-type parasites and tried to reveal overexpressed genes that may result in enhanced virulence of *ΔNcACBP*. Among 8 genes with a 6-fold higher increase in *ΔNcACBP* parasites, we observed that NCLIV_033380, NCLIV_069820, NCLIV_019580, and NCLIV_020100 are presumed to be involved in cellular membrane biogenesis, while there are still several genes with unknown protein domains and functions such as NCLIV_052460, NCLIV_020100, NCLIV_023980, and NCLIV_002870. These four proteins have no signal peptides, indicative of the low possibility of secretion into PV and host cells to regulate host-parasite interaction. In addition, we also analyzed 25 genes with a 4–6-fold transcriptional increase and also found that most of these genes may be involved in membrane biogenesis. However, transcriptome sequencing did not identify any genes related to known *Neospora* virulence factors, such as ROP5, ROP16 and GRA17.

Reportedly, lipids as an important pathogenic factor can help parasites escape the host immune response and cause diseases [5, 49]. In our study, the TAG abundance was increased in the *ΔNcACBP* strains compared with that in the wild-type strain. Moreover, an increased amount of neutral lipids derived from host lipid bodies was observed surrounding the *ΔNcACBP* strains compared with that in the wild-type strain. Although these lipid bodies were mainly detected under experimental conditions, they may be relevant physiologically and involved in nutrient uptake and delivery to the parasite’s cytoplasm potentially. Lipid body formation is closely linked to the biosynthesis of neutral lipids, such as sterols and TAGs that are packaged in the lipid droplet core [50, 51]. TAG is formed by redundant non-esterified free fatty acids, which are cytotoxic at high concentration, in a self-protective manner [52]. In addition, our laboratory has previously shown that disruption of ACBP alone in *Toxoplasma* did not affect the growth ability and intracellular replication, which is the same as NcACBP knockout phenotypes *in vitro* [24]. However, TgACBP disruption did not affect the virulence to mice while NcACBP knockout enhanced the pathogenicity to mice. Notably, TgACBP and TgSCP2 double disruption reduced the TAG overall abundance while NcACBP knockout resulted in the accumulation of TAG. Therefore, we hypothesize that one possibility of the virulence difference lies in the metabolic regulation mechanism of TAG between *Toxoplasma* and *Neospora*. TAG could provide acyl skeleton for phospholipids and other many kinds of lipid derivatives, which can be involved in membrane synthesis. Transcriptional levels of many genes related to membrane biogenesis have been identified, indicating that NcACBP disruption may lead to membrane biogenesis disorder and thus affect the immunological recognition of specific antigens in parasite membranes by the host. In this perspective, we should apply lipidomics to reveal lipid components in cellular membranes of *ΔNcACBP* parasites and pay more attention to the immunological response of host cells to *ΔNcACBP* infection in future work.

Stable isotope labeling and metabolomic analysis showed that loss of NcACBP did not significantly alter the total abundance of fatty acids or affect the synthesis of fatty acids, nor did it impair the synthesis of unsaturated long-chain fatty acids or the ability to salvage short-chain fatty acids from the host. Furthermore, because U-^13^C-glucose labeled fatty acids were synthesized by the FASII pathway, there may be alternate substrates for fatty acid synthesis, and the sources of fatty acids are likely complicated and diverse. As intracellular pathogens acquire essential non-diffusible host metabolites [53], perhaps the fatty acid uptake of NcACBP-deficient parasites is increased in host cells. However, we hypothesize that proteins functionally compensatory to NcACBP are expressed to compensate for fatty acid metabolism in NcACBP-deficient parasites. The results of the present studies strongly suggest that in addition to the essential fatty acid synthesis pathways (FASI, FASII, and FAE), intracellular tachyzoites can salvage fatty acids from the host and the surrounding environment in a fine-tuned manner to meet their own needs [4, 35, 54]. Since there are few studies on fatty acid biosynthesis and metabolism in *N. caninum*, we propose that the fatty acid biosynthesis and metabolic pathways in *N. caninum* may be similar to those in *T. gondii* and *P. falciparum*, based on our results and previous studies [35, 54]. Acetyl-CoA is a key metabolite involved in the TCA cycle, fatty acid synthesis, fatty acid elongation, etc. The apicoplast FASII pathway generates its own pool of acetyl-CoA, mitochondria generate acetyl-CoA for the FASII pathway in the cytosol, and the elongation pathway in the ER relies on the TCA cycle. In addition, fatty acids and lipids scavenged from the host may be a possible redundant source for parasites.

## Conclusions

We demonstrated that NcACBP is not an essential gene during the tachyzoites stage and is present as a short ACBP dispersed in the cytoplasm. Genetic ablation of NcACBP did not impair the survival and growth phenotype of *N. caninum* but enhanced its pathogenicity in mice. This deletion did not affect the overall fatty acid composition but did modify the abundance of TAG and loss of NcACBP resulted in global changes in the expression of multiple genes.

## Supplementary information

**Additional file 1: Table S1.** The sequence of primers. **Table S2.** The differentially regulated genes between Nc-1 and *ΔNcACBP* strains.

## Data Availability

Data supporting the conclusions of this article are included within the article and its additional file.

## Abbreviations

CRISPR/Cas9: clustered regularly interspaced short palindromic repeats/CRISPR associated protein 9
FAS: fatty acid synthesis
FAE: fatty acid elongation
FABP: fatty acid binding protein
SCP2: sterol carrier protein 2
ER: endoplasmic reticulum
PL: phospholipids
LPA: lysophosphatidic acid
LCFA: long-chain fatty acid
VLCFA: very long-chain fatty acid
FBS: fetal bovine serum
BSA: bovine serum albumin
UTR: untranslated region
ORF: open reading frame
GFP: green fluorescence protein
GC-MS: gas chromatography-mass spectrometry
TLC: thin layer chromatography
TAG: triacylglycerol
KO: knockout
dpi: day post-infection
FDR: false discovery rate
KEGG: Kyoto Encyclopedia of Genes and Genomes
GO: Gene Ontology
TCA: tricarboxylic acid cycle
NLS: nuclear localization signal
DHFR: dihydrofolate reductase
