# Supplementary material for: Functional characterization of acyl-CoA binding protein in Neospora caninum
Source: Parasit Vectors. 2020 Feb 18;13:85. doi: 10.1186/s13071-020-3967-9 (PMC7029560; doi:10.1186/s13071-020-3967-9)
Supplement: Supplementary file 1 — Additional file 1: Table S1. The sequence of primers. Table S2. The differentially regulated genes between Nc-1 and ΔNcACBP strains. [file 13071_2020_3967_MOESM1_ESM.docx]

**Additional file 1: Table S1. Primer sequences**

| Primer | Sequence |
| --- | --- |
| F1 | ATGGCCTCGCAAGAAGAATTCGAGAGGGCCG |
| R1 | CTACGCGCTCTTCTCGCGCCACGCGGGCTGA |
| F2 | TAGGGCGAATTGGGTACCCAACCCTCTGCGGATTCT |
| R2 | AAAGTGTTCACCTGGCTTCAGCACATCCG |
| F3 | GGATCCACTAGAGATATCCACTGAGAGAACGGTGTCCTT |
| R3 | TATCACTGAGGGAACGGGGTGGGTAG |
| F4 | ATGGCCTCGCAAGAAGAATT |
| R4 | GCCACGCGGGCTGAATTCTA |
| F5 | GGTTTGCCGGCGCCTTCGTC |
| R5 | GGCGGAGCGTCGGGGGATG |

**Additional file 1: Table S2** The differentially regulated genes between Nc-1 and *ΔNcACBP* strains

| Gene ID | Name | log2FoldChange |
| --- | --- | --- |
| NCLIV_017951 | unspecified product | -5.9542 |
| NCLIV_009480 | unspecified product | -5.39 |
| NCLIV_033120 | GM16080, related | -5.2435 |
| NCLIV_006820 | conserved hypothetical protein | -5.2173 |
| NCLIV_005480 | hypothetical protein | -5.1359 |
| NCLIV_049140 | hypothetical protein | -5.1359 |
| NCLIV_052800 | SRS domain-containing protein | -5.0316 |
| NCLIV_017951 | unspecified product | -5.9542 |
| NCLIV_061800 | putative protein disulfide-isomerase | -3.8612 |
| NCLIV_033070 | putative MORN repeat-containing protein | 6.5909 |
| NCLIV_034420 | SRS domain-containing protein | 6.0376 |
| NCLIV_035020 | putative Dpy-30 motif containing protein | 4.9809 |
